# Ovarian female adnexal tumor of probable Wolffian origin – Case report

**DOI:** 10.1515/med-2021-0306

**Published:** 2021-06-23

**Authors:** Ljiljana Vučković, Aleksandra Klisic, Mirjana Miladinović

**Affiliations:** Clinical Center of Montenegro, Department of Pathology, University of Montenegro-Faculty of Medicine, Podgorica, Montenegro; Center for Laboratory Diagnostics, Primary Health Care Center, University of Montenegro-Faculty of Medicine, Trg Nikole Kovacevica 6, 81000 Podgorica, Montenegro

**Keywords:** ovarian tumor, FATWO, immunohistohemistry

## Abstract

**Background:**

During embryonic development in women, a regression of temporary embryonic structures – mesonephric (Wolffian) ducts occurs. Adnexal tumors of Wolffian duct origin (FATWO) are rare.

**Case report:**

We presented the case of a 64-year-old female patient who was diagnosed with FATWO. After the surgical treatment, the uterus with bilateral adnexal structures was submitted for histopathological analysis. The left ovary was occupied by a tumor measuring 80 × 60 × 50 mm, with smooth, shiny, whitish surface. Tumor cells were medium-sized, relatively uniform, round, and polygonal, with eosinophilic cytoplasm and centrally laid nucleus with fine chromatin, organized into solid, trabecular, and tubular formations. Tumor cells were positive for pancytokeratin (CK), CK7, CD10, neuron-specific enolase (NSE), synaptophysin, calretinin, progesterone, estrogen, and epithelial membrane antigen (EMA).

**Conclusion:**

This case adds a report of a rare tumor to the literature. We must think of it in the differential diagnostic algorithm to make an accurate diagnosis for selecting the best treatment modality.

## 1 Introduction

During embryonic development in women, a regression of temporary embryonic structures – mesonephric (Wolffian) ducts occurs. However, the remains of these ducts can be found postnatally in the vagina, cervix, and uterine corpus, as well as in the adnexal structures, mesosalpinx, broad ligament, and peritoneum [1,2]. Adnexal tumors of Wolffian duct origin (FATWO) in the female population belong to the group of epithelial tumors that are rare. They were first described in 1973 in the article by Kariminejad and Scully [3]. So far, less than 100 cases have been reported in the literature in the English language [4]. These are tumors of benign biological potential, with a nonspecific clinical picture and radiological characteristics [2]. These tumors most commonly occur in the uterine parametrium and tubes, although cases of their occurrence in the ovaries have been described [5]. FATWO most commonly occurs around the age of 50, and in more than half of the cases, it is accidentally found during regular gynecological examinations [6]. From the histological point of view, the tumor tissue shows great intra- and intertumor heterogeneity. Tumor cells are usually small- or medium-sized, round, oval, or spindle-shaped. A large number of different histological patterns can be seen: solid, tubular, sieve-like pattern, trabecular, microcystic, or a combination of the above. There is no specific immunohistochemical staining to diagnose this tumor. The pathogenesis and molecular characteristics of this tumor are not clear enough. Given the above, the histopathological diagnosis of FATWO presents a major challenge [2,6]. In relation to this tumor, several terms have been used in the literature – Wolffian adnexal tumor, Wolffian adenoma, and retiform Wolffian adenoma. The World Health Organization has proposed the name Wolffian tumor in its classification of female reproductive system tumors [7].

This study presents the case of a 64-year-old patient who was diagnosed with FATWO during a routine gynecological examination.

## 2 Case report

During the routine gynecological examination of a 64-year-old patient, a tumor mass of the left ovary measuring 80 mm was found. Symptoms were vague, and subsequently, the patient complained of bloating and discomfort in the pelvis area. The patient was in menopause for 12 years and had two births. The patient was in good general condition and was not treated for other diseases. Transabdominal and transvaginal ultrasonography were performed, which showed the solid homogenous tumor in the projection of the left ovary. The result of the cross-sectional CT scan confirmed the diagnosis of ovarian tumor. According to the results of radiological diagnostic procedures that suggested left ovarian tumor mass, surgical removal was indicated. In preoperative preparation, laboratory parameters including tumor markers CA-125, CA 15-3, CA 19-9, and CEA were within the reference values. Ultrasound examination of the abdomen and X-ray of the lungs showed no changes in other organs. Exploratory laparotomy was performed, with a hysterectomy and bilateral adnexectomy.

After the surgical treatment, the uterus with bilateral adnexal structures was submitted for histopathological analysis. The left ovary was occupied by a tumor nodule measuring 80 × 60 × 50 mm with a smooth, shiny, whitish, nodular surface. The serial cross-sections were partly solid and partly cystic-altered tumor tissue, yellowish-white in color, with a homogeneous structure and medium-firm consistency. The remains of ovarian tissue were recognized peripherally in the tumor node. The tube next to the tumor node was of appropriate macroscopic structure. No significant macroscopic changes were found in the cervix, uterine corpus, as well as in the right ovary and right tube.

**Figure 1:**
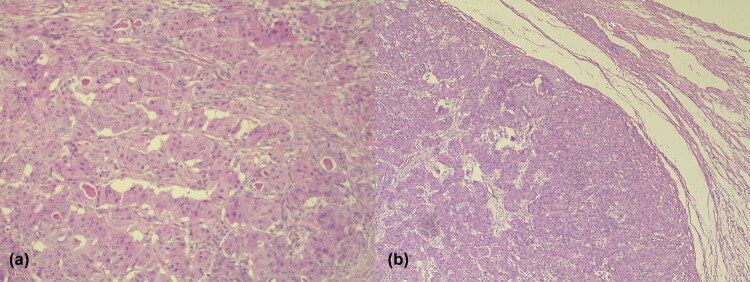
(a) Eosinophilic, colloid-like content in the lumen of tubular formation of the tumor cells (HE, ×10). (b) Expansive tumor growth toward the ovarian cortex (HE, ×5).

Tumor tissue was composed of medium-sized, relatively uniform, round, and polygonal cells, eosinophilic cytoplasm and centrally laid nucleus, fine chromatin, and some clearly visible nucleoli. Tumor cells were organized into solid, trabecular, and tubular formations. Dense, eosinophilic, colloid-like content was observed in the lumen of tubular formations. The tumor stroma was for the most part very scarce. In minor areas, the stroma was more abundant, with myxoid appearance. No necrosis was found in the tumor tissue. Mitoses were very rare (focal 1–2 mitoses/10HPF). Tumor cells did not infiltrate the ovarian connective capsule (Figure 1).

Immunohistochemical analysis of tumor tissue was performed. Tumor cells were positive for pancytokeratin (CK), CK7, CD10, and synaptophysin. Individual tumor cells were positive for calretinin, progesterone, estrogen, epithelial membrane antigen (EMA), and neuron-specific enolase (NSE).

**Figure 2:**
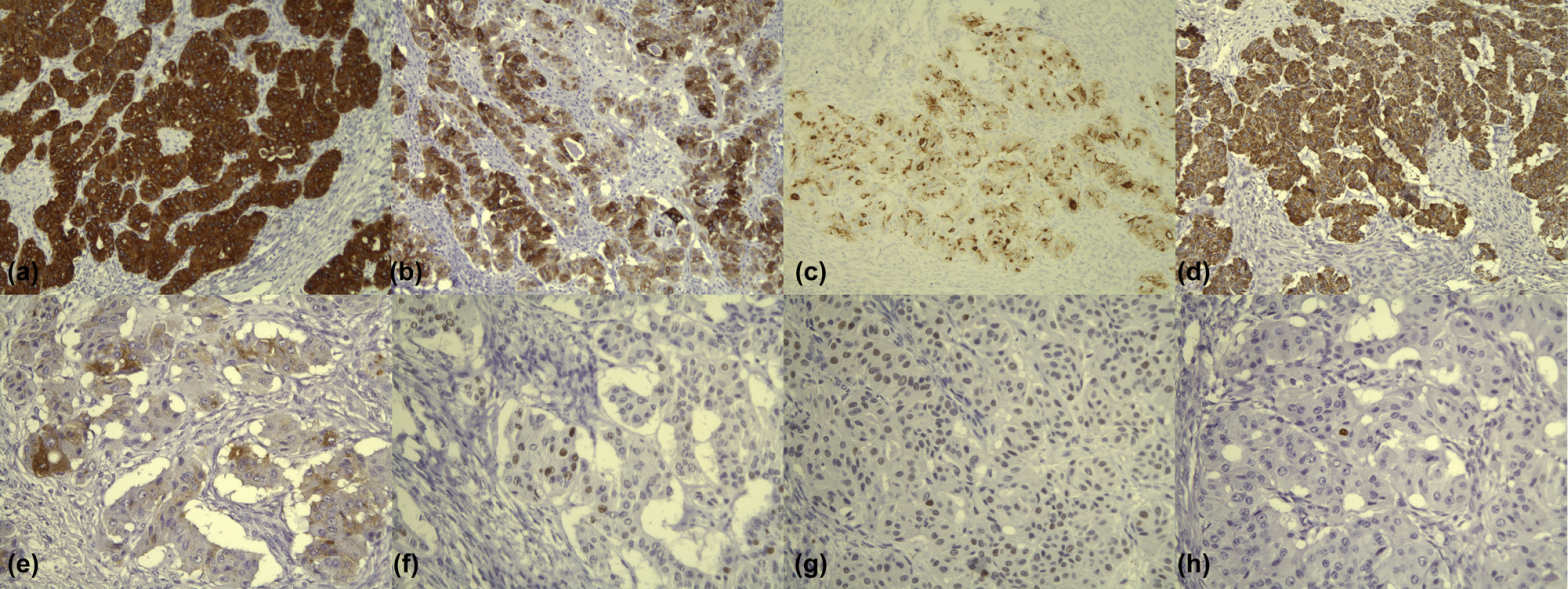
Immunohistochemical analysis of the tumor. (a) CK, ×10; (b) CK7, ×10; (c) CD10, ×10; (d) synaptophisin, ×10; (e) EMA, ×20; (f) estrogen, ×20; (g) progesterone, ×20; (h) Ki67, ×20.

Tumor cells were negative for inhibin, CD20, CDX2, Wilms tumor gene 1 (WT1), CD56, CD125, thyroid transcription factor 1 (TTF1), paired box 8 (PAX8), p16, mammaglobin, GATA binding protein 3 (GATA3), gross cystic disease fluid protein 15 (GCDFP15), and chromogranin. Ki67 expression was less than 1% (Figure 2).

Based on the morphology of the tumor tissue and the results of immunohistochemical analysis, the diagnosis of FATWO was made. Considering it is a tumor with substantial malignant potential, the patient was advised to have check-ups, which included regular clinical examinations, abdominal ultrasound, and X-rays of the lungs.

One year after the operation, there were no signs of disease recurrence or distant metastases.


**Informed consent:** Prior to documenting the FATWO case report, informed patient consent was obtained in accordance with the institution’s recommendations.

## 3 Discussion

In this study, we presented a 64-year-old patient, where a FATWO of the left ovary, 80 mm in size, was detected accidentally during a routine clinical examination. Preoperative values of serum tumor markers (CA-125, CA 15-3, CA 19-9, and CEA) were within the reference values. The tumor diagnosis was done based on the pathohistological analysis of operative material.

Most often, macroscopically, it is an encapsulated, solid, or solid-cystic tumor. In serial sections, the tumor tissue is grayish-yellow or light brown in color. Bleeding and necrosis may be present in larger tumors [2,6].

Histologically, this tumor can show great intra-tumor and inter-tumor heterogeneity and different histological patterns. In most cases, a combination of multiple histological patterns is present. Histological patterns can be solid, tubular, sieve-like, trabecular, and microcystic. Tumor cells are small- or medium-sized, epithelial, or spindle-shaped. The nuclei are small- to medium-sized, round, oval, or spindle-shaped; some tumor cells have prominent nuclei. Cellular atypia is minimal. Mitotic activity is low. PAS-positive eosinophilic secretion can be seen within the lumen of some tubules, especially cystic spaces. The stroma varies from a delicate and sparse network of reticulin fibers to large areas of hyalinized, slightly cellular stroma [2,4,6].

Shalaby and Shenoy [2] indicate that there is no single specific immunohistochemical marker for the diagnosis of FATWO. They state that immunopositivity for CK (100%), CAM5.2 (100%), CK7 (88%), CK903 (17%), CK8, CK18, CD10, calretinin (91%), inhibin A (68%), and vimentin (100%) indicates FATWO.

The tumor is usually negative for EMA, S100, actin, CD15, human bone marrow endothelial marker-1 (HBME-1), and CK20. Staining for chromogranin, synaptophysin, and NSE is usually weakly positive. They describe the variable expression of estrogen and progesterone receptors, androgen receptor, WT1, and CD117 (c-Kit) [2].

Hong et al. [8] indicated that the histology and immunohistochemical profile are crucial for the diagnosis of this tumor. In morphological analysis, it is important to keep in mind that the tumor is characterized by heterogeneity. Solid, tubular, and cribriform patterns are often present, as well as glandular structures whose lumens contain eosinophilic content. IHC panel that includes CD10, estrogen, progesterone, CK7, EMA positive cells, carcinoembryonic antigen (CEA), inhibin, CD99, calretinin, WT1, PAX8, and CK20 negative cells indicates FATWO. Positive CD10 expression and PAX8 negative expression are considered to be the most helpful markers in the diagnosis of FATWO [8].

Hubner et al. [5] indicated the importance of CD10, CK, and CK7 positivity in the diagnosis of this tumor. They believe that vimentin, calretinin, and inhibin can be positive (markers of sex cord-stromal tumors) in most cases, while positivity to steroid receptors is less common [5].

Liu et al. [9] stated that FATWO is positive for calretinin, CAM5.2, CK8, CK18, CD10, CK7, and vimentin and negative for EMA, S100, actin, CD15, HBME-1, and CK20. Chromogranin, synaptophysin, and NSE are usually weakly positive. Estrogen and progesterone show uncertain immunohistochemical behavior. This group of authors suggests that CD56 expression could help to determine the biological potential of this tumor. Nevertheless, these data certainly require further consideration [9].

The main differential diagnosis of FATWO includes carcinomas (endometrioid, serous, clear cell), sex cord-stromal ovarian tumors, and nongynecological metastatic tumors [8]. When it comes to FATWO morphology, a combination of multiple histological patterns is often present which helps in the differential diagnosis of this tumor when compared with others [2].

In ovarian cancers, unlike FATWO, cellular atypia, nuclear pleomorphism, and numerous mitoses are present. Squamous metaplasia and mucin production are often observed in endometrioid carcinoma. Carcinomas are positive for a wide range of cytokeratins, EMA, and WT1, while negative for calretinin and inhibin [2,4,6,8].

Sertoli-Leydig cell tumors may have strong morphological similarities to FATWO. The presence of a sieve-like arrangement of tumor cells and the absence of Leydig cells are useful when it comes to FATWO diagnosis. In addition to that, Sertoli-Leydig tumors may be accompanied by endocrine symptomatology that is not typical of FATWO. Granulosa cell tumor (GCT) is morphologically characterized by grooved nuclei and sparse cytoplasm of tumor cells. GCT may also be accompanied by endocrine symptomatology. GCTs are usually negative for CK7 and positive for CK in 30 to 37% of cases. Recent research suggests that steroidogenic factor 1 (SF-1) may help distinguish FATWO from sex cord-stromal tumors. FATWO has been consistently negative for this marker in previous studies, while it was positive in most sex cord-stromal tumors. In sex cord-stromal tumors, inhibin-α is usually diffusely positive, in contrast to focal positivity in FATWO [2,6,7].

Mirković and co-authors [10] analyzed mutations in a series of seven diagnosed FATWO cases. Mutations were analyzed using a 300-gene panel. They concluded that there were no common mutations. In the analyzed series, they did not find KRAS/NRAS mutation (characteristic of mesonephric cancer), then DICERI mutation (characteristic of Sertoli-Leydig tumor), PTEN, PIK3CA, KRAS, and CTNNB1 mutations (characteristic of endometrial cancer). They proved the existence of the KMT2D mutation, which is still of unclear biological significance. They suggest larger gene panels or whole-exome sequencing [10].

Recent research suggests that most FATWOs are of benign biological potential, but tumors of malignant biological potential have also been shown, with metastases in the pelvis, abdomen (most commonly liver), and chest (lung). Overall, the literature supports the fact that approximately one-fifth of FATWOs is malignant. The average time for relapses in previous articles was 48 months (range from 13 to 96 months). The malignant potential of FATWO has not been established by its molecular and immunohistochemical properties. The presence of necrosis, capsular invasion, a large number of mitoses, cellular pleomorphism, immunohistochemical positivity to CD117, and probably over-expression of Ki67 are currently known properties of the malignant variant of FATWO. However, a clear malignant biological potential is determined by recurrences and the appearance of metastases.

Complete surgical resection with hysterectomy, bilateral adnexectomy, and tumor removal are considered as the most effective therapies for FATWO. Chemotherapy and radiation therapy have a controversial role in the treatment of recurrent and malignant FATWO. Targeted therapies (use of tyrosine kinase inhibitors) are possible potential methods of treatment [2,7,8,9,11].

Vitale et al. [12] gathered all the evidence reported in the literature about gynecological cancers in the elderly (i.e. in adults older than 65) and concluded that in elderly patients who often have comorbidities, the extent of surgery and the aggressiveness of chemotherapy should be tailored to the individual’s extend of disease, symptoms, overall health, and life goals [12]. The results of previous studies [13,14] suggest that the robotic laparoscopic treatment of gynecological cancer is a safe and feasible technique, comparing to traditional laparoscopic surgery with advantages in terms of precision and reduction of intraoperative bleeding. This is especially related to cases of an early stage of ovarian cancer since it gives equally good results as surgical laparoscopy (comparing the perioperative and gynecologic-oncologic outcomes) [14]. The majority of patients with ovarian cancer, which is the most frequent ovarian neoplasm, achieved complete remission after the first line of chemotherapy and, subsequently, present a relapse which, in most cases, leads to death [15]. Adequate understanding of the underlying molecular mechanism of chemo-resistance may lead to identifying the possible biomarkers that could be applied to plan effective personalized therapies [16]. It seems that initial clinical response is due primarily to the therapeutic efficacy of chemotherapy against differentiated cancer cells, whereas the high rate of recurrence is thought to be due to remaining drug-resistant cells, biologically distinct, identified as cancer stem cells [15]. Otherwise, ovarian cancer initiation and progression may depend on the surrounding microenvironment with stromal and immune cells [16]. Immune cells not only protect the host against tumor cells but also shape tumor immunogenicity, which stress the dual host-protective and tumor-promoting actions of immunity on developing tumors [17].

This mechanism proceeds sequentially through three distinct phases termed “elimination” (of tumor cells by immune cells), “equilibrium” (immune cells prevents tumor cells overgrowth), and “escape” (tumor cells have acquired the ability to avoid the response of immune cells). This evidence comes from diverse epidemiologic and clinical data comprising: evidence of spontaneous antitumor immune response and its association with longer survival in a proportion of patients with ovarian cancer; evidence of tumor immune evasion mechanisms and their association with short survival in some patients with ovarian cancer; and pilot data supporting the efficacy of immune therapy [17,18].

In our case, the tumor is considered as FATWO according to localization, morphological characteristics, and immunohistochemical profile.

## 4 Conclusion

This case adds a report of a rare tumor to the literature. We must think of it in the differential diagnostic algorithm to make an accurate diagnosis for selecting the best treatment modality.
